# The outcome of pulmonary hypertension and its association with pulmonary artery dilatation

**DOI:** 10.1007/s12471-020-01467-1

**Published:** 2020-07-16

**Authors:** A. L. Duijnhouwer, J. Lemmers, J. Smit, J. van Haren-Willems, H. Knaapen-Hans, T. ten Cate, W. Hagmolen of ten Have, M.-J. de Boer, J. Roos-Hesselink, M. Vonk, A. van Dijk

**Affiliations:** 1 Department of Cardiology and Radboudumc Centre for Pulmonary Hypertension, Radboud University Medical Centre, Nijmegen, The Netherlands; 2 Department of Rheumatic Diseases and Radboudumc Centre for Pulmonary Hypertension, Radboud University Medical Centre, Nijmegen, The Netherlands; 3 Department of Pulmonology and Radboudumc Centre for Pulmonary Hypertension, Radboud University Medical Centre, Nijmegen, The Netherlands; 4 Department of Cardiology, Erasmus Medical Centre, Rotterdam, The Netherlands

**Keywords:** Hypertension, Pulmonary, Pulmonary artery, Dilatation

## Abstract

**Background:**

Pulmonary artery (PA) dilatation is often seen in pulmonary hypertension (PH) and is considered a long-term consequence of elevated pressure. The PA dilates over time and therefore may reflect disease severity and duration. Survival is related to the stage of the disease at the time of diagnosis and therefore PA diameter might be used to predict prognosis. This study evaluates the outcome of patients with pulmonary arterial hypertension (PAH) and chronic thrombo-embolic pulmonary hypertension (CTEPH) and investigates whether PA diameter at the time of diagnosis is associated with mortality.

**Methods:**

Patients visiting an outpatient clinic of a tertiary centre between 2004 and 2018 with a cardiac catheterisation confirmed diagnosis of PAH or CTEPH and a CT scan available for PA diameter measurement were included. PA diameter and established predictors of survival were collected (New York Heart Association (NYHA) class, N‑terminal pro-brain natriuretic peptide (NT-proBNP) level and 6‑min walking distance (6MWD)).

**Results:**

In total 217 patients were included (69% female, 71% NYHA class ≥III). During a median follow-up of 50 (22–92) months, 54% of the patients died. Overall survival was 87% at 1 year, 70% at 3 years and 58% at 5 years. The mean PA diameter was 34.2 ± 6.2 mm and was not significantly different among all the diagnosis groups. We found a weak correlation between PA diameter and mean PA pressure ( *r* = 0.23, *p* < 0.001). Male sex, higher age, shorter 6MWD and higher NT-proBNP level were independently associated with mortality, but PA diameter was not.

**Conclusion:**

The prognosis of PAH and CTEPH is still poor. Known predictors of survival were confirmed, but PA diameter at diagnosis was not associated with survival in PAH or CTEPH patients.

## What’s new?

- Pulmonary artery diameter is not associated with prognosis in pulmonary arterial hypertension (PAH) and in chronic thrombo-embolic pulmonary hypertension. Six-minute walking distance and N‑terminal pro-brain natriuretic peptide level were confirmed as prognostic parameters.
- Patients suspected for PAH are referred at a late stage of the disease.
- Prognosis seems not to have improved in the last 5 years, despite new treatment options.
- Patients with PAH associated with connective tissue disease still have the worst prognosis.

## Introduction

Pulmonary hypertension (PH) is a haemodynamic and pathophysiological condition defined as an elevated mean pulmonary arterial pressure (PAP) ≥25 mm Hg. The World Health Organization divides PH into five groups based on aetiology. The general term PH can be used to describe all these groups together. The term pulmonary arterial hypertension (PAH) is used to describe patients with PH with normal pulmonary capillary wedge pressure (≤15 mm Hg) and elevated pulmonary vascular resistance (>3 WU) in the absence of other causes of precapillary PH such as that due to lung diseases. PAH and chronic thrombo-embolic pulmonary hypertension (CTEPH) are considered to be rare diseases with an estimated incidence of 2.2–7.6 patients per one million adults [1–3]. Due to this low incidence, evaluation of survival and mortality is challenging [3, 4].

Some recent studies have shown that the combination of New York Heart Association (NYHA) functional class, the level of N‑terminal pro-brain natriuretic peptide (NT-proBNP) and the 6‑min walking distance (6MWD) are reasonably good independent non-invasive predictors of mortality in patients with PAH [5]. Although these parameters have limitations (6MWD is of limited value in patients with impaired mobility) to date, they are the best predictors available and are currently used to assess treatment response [6].

Pulmonary artery (PA) dilatation can be used to help identify PH patients by echocardiographic screening, cardiac magnetic resonance imaging or thoracic computed tomography (CT) [7–9]. Some authors have suggested that PA dilatation is a predictor of an unexpected death, implying that increased PA wall stress might lead to fatal PA rupture or dissection. Up to now, no association of PA dilatation with all-cause mortality has been found [10–12].

Theoretically, it seems logical to assume that the PA diameter is larger in patients in whom the PAP has been elevated for a longer time, suggesting that patients with a larger PA at diagnosis have had their disease longer and therefore have a poorer prognosis [13]. New treatment options have become available and have been shown to improve clinical status and survival. Monitoring the effects of these new treatment options has become more relevant [14].

This study aims to evaluate the survival of patients with PAH and CTEPH in the current era and to investigate whether PA diameter at the time of diagnosis is associated with mortality.

## Methods

All patients visiting a single pulmonary hypertension expert centre between January 2004 and November 2018 were eligible for inclusion. Inclusion criteria were a right heart catheterisation confirming the diagnosis of PAH or CTEPH and a thoracic CT scan on which the PA diameter could be measured.

The date of the first right heart catheterisation was considered to be the date of diagnosis. For inclusion, data on NYHA classification, NT-proBNP level or the 6MWD were required.

**Fig. 1:**
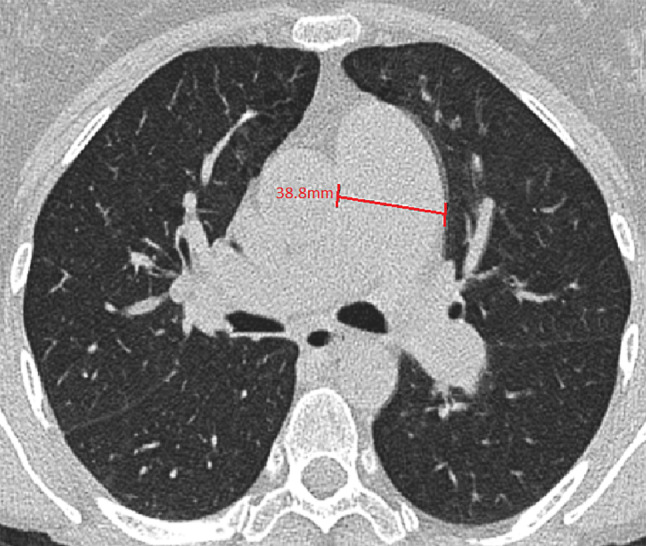
Example of a measurement of the main pulmonary artery in the transverse plane at the level of the main pulmonary artery on CT scan

The CT scans were performed for different reasons and therefore not all of them included administration of a contrast medium. The main PA diameter was measured perpendicular to the PA wall in a transversal view at the level of the aorta at which level the right PA was also visible (Fig. 1).

PA dilatation was defined as a diameter ≥29 mm for men and ≥27 mm for women, following the upper limit of normal (90th percentile) proposed by the Framingham Heart study [15].

In concordance with other studies, a composite risk score was calculated to compensate for missing variables [5, 6, 16, 17]. NYHA class ≥III, a 6MWD <440 m or a NT-proBNP level >300 pg/ml were considered high-risk. To compute the risk score, the number of high-risk factors was counted and divided by the number of available variables per patient, so the risk score could be between 1 (high risk) and zero (low risk). Information on the vital status of all participants was obtained from the municipal personal records database (*Gemeentelijke Basis Administratie*) of the Netherlands.

### Data analysis

Continuous data were expressed as a mean ± SD when normally distributed, otherwise as a median and interquartile range. Comparisons among PAH and CTEPH were made using ANOVA tests for continuous, normally distributed data and the Kruskal-Wallis test for the continuous, not normally distributed data. The distribution of categorical and binary variables was tested with the chi-square or the Fisher exact test. Post hoc comparisons were done using the Bonferroni method. *A p* value ≤0.05 was considered significant. We used Pearson’s rho tests and linear regression to find possible associations between PA diameter and other variables. Patients who underwent lung transplantation were censored at the time of the procedure. The survival rates were calculated with the Kaplan-Meier method.

We used a univariate Cox proportional hazards regression to identify risk factors for survival. For known prognostic factors of mortality a *p* value <0.10 was considered statistically significant.

## Results

A total of 217 patients were included, 69% of them female, with a median age of 65 years. The baseline characteristics of all patients are shown in Tab. 1.

**Table 1:** Baseline characteristics

Characteristics	Total	IPAH	CTD-PH	CHD-PH	CTEPH
Patients, *n*	217	52	103	17	42
Age (years)	65 (51–73)	64 (47–76)	65 (55–72)	48 (35–66)	67 (58–75)
Female (%)	69	77	75	65	50 ^a^
Height (cm)	167 ± 9	166 ± 10	167 ± 9	167 ± 10	171 ± 9
Weight (kg)	71 (62–82)	73.5 (66–82)	68 (61–81)	59 (55–80)	74 (68–89)
BSA	1.80 (1.67–1.94)	1.81 (1.69–1.93)	1.76 (1.67–1.93)	1.66 (1.55–1.96)	1.88 (1.74–2.10)
*NYHA FC (%)*					
I	3	2	2	6	6
II	26	18	26	38	36
III	58	59	59	50	55
IV	13	22	13	6	3
PA diameter (mm)	34 (31–38)	36 (31–41)	33 (30–37)	37 (30–47)	34 (32–38)
PA dilatation (%)	95	98	90	100	98
Mean PAP (mm Hg)	41 (31–52)	48.5 (38–54)	37 (28–49)	41.5 (32–49)	40.5 (31–47)
NT-proBNP	1587 (341–4048)	2896 (610–6223)	1090 (295–3837)	341 (249–3975)	1550 (350–2696)
6MWD (m)	340 ± 120	302 ± 143	356 ± 114	315 ± 69	367 ± 91
Death (%)	54	52	67	41	33
Follow-up time (days)	1513 (681–2809)	1414 (620–2504)	1264 (645–2393)	2863 (1363–9599)	1880 (845–3187)
Follow-up time (months)	50 (22–92)	46 (20–82)	42 (21–79)	94 (45–315)	62 (28–105)

All values with ± are means with standard deviation. All values with (#–#) are medians with interquartile range.

*IPAH* idiopathic pulmonary arterial hypertension, *CTD-PH* pulmonary hypertension associated with connective tissue disease, *CHD-PH* pulmonary hypertension associated with congenital heart disease, *CTEPH* chronic thromboembolic pulmonary hypertension, *BSA* body surface area, *NYHA FC* New York Heart Association functional class, *PA* pulmonary artery, *PAP* pulmonary artery pressure, *NT-proBNP* N-terminal pro-brain natriuretic peptide, *6MWD* 6-min walking distance

^a^ Significantly different in comparison with the other groups

PH associated with connective tissue disease (CTD-PH) was the largest group (*n* = 103, 47.5%), idiopathic PAH (IPAH) 24%, CTEPH 19.4%, congenital heart disease (CHD)-PH 7.8%, heritable PAH 0.9% and drug-induced PAH 0.5%.

The largest proportion (71%) of our population was in NYHA functional class ≥ III at inclusion.

### Group comparison

In 93 patients, all variables were available within our chosen time range. Comparison between these patients and the other 124 with partly missing data revealed no statistically significant differences in any of the variables apart from PA diameter. In the 93 patients with complete data, a mean PA diameter of 34.0 ± 5.4 mm was observed; the other group had a mean of 35.6 ± 5.4 mm, *p* = 0.035. Of the total of 217 patients, 95% had a dilated PA. The mean calculated risk score for the 93 patients with complete data was 0.77 ± 0.31 versus 0.78 ± 0.38 for the remaining group.

### PA diameter

The mean PA diameter was 34.2 ± 6.2 mm, indexed PA diameter (PA diameter (mm)/body surface area (BSA)) 19.2 ± 3.9 mm/m^2^. PA dilatation was observed in 95% of the women and in 93% of the men. There was no statistically significant difference in PA diameter between PAH and CTEPH, among the NYHA functional classes or the composite risk score groups.

Analysis of the relation between PA dilatation and the mean PAP revealed a weak but statistically significant correlation (*r* = 0.23, *p* < 0.001).

### Survival

The median follow-up time of all patients was 1513 days. No patients were lost to follow-up. Two patients underwent lung transplantation and 118 patients (54.4%) died.

The cumulative survival of all patients was 87% at 1 year, 70% at 3 years and 58% at 5 years, with a total of 85 deaths during 5 years of follow-up and 93 patients still being followed up at 5 years.

**Fig. 2:**
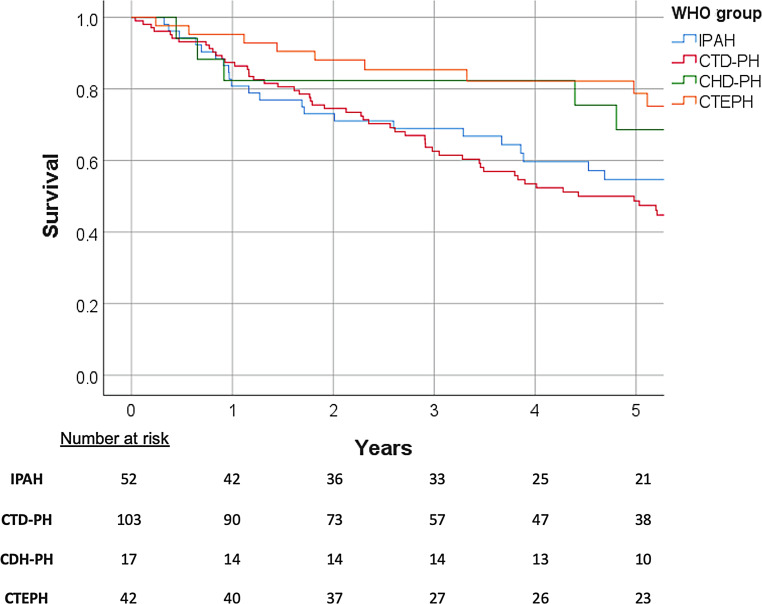
Survival curves of the four investigated pulmonary hypertension groups. *IPAH* idiopathic pulmonary arterial hypertension, *CTD-PH* pulmonary hypertension associated with connective tissue disease, *CHD-PH* pulmonary hypertension associated with congenital heart disease, *CTEPH* chronic thromboembolic pulmonary hypertension

Fig. 2 shows the survival rates of the separate groups. The 5‑year survival for IPAH is 55%, for CTD-PH 47%, for CHD-PH 69% and for CTEPH 79%.

**Fig. 3:**
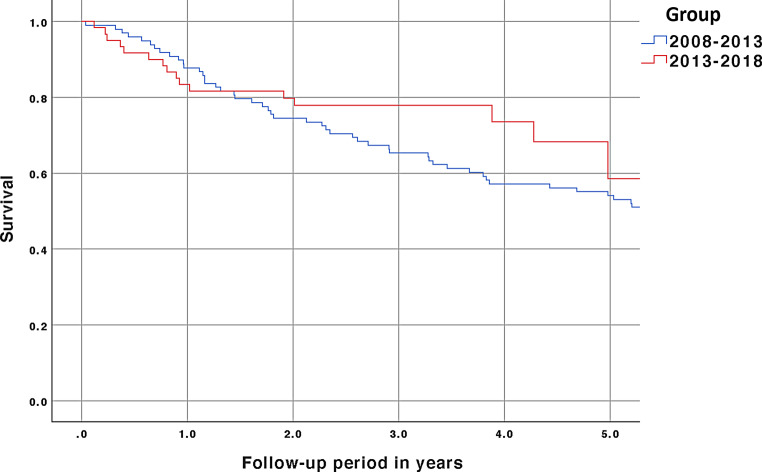
Comparison of survival curves of patients diagnosed in the periods 2008–2013 and 2013–2018

Comparison of survival rates between patients diagnosed from 2008–2013 and 2013–2018 yielded no significant difference (Fig. 3). During the period 2008–2013, 46% patients died and during the period 2013–2018 39% of the patients at risk died. These proportions were not statistically different (*p* = 0.28).

### Factors associated with prognosis

**Fig. 4:**
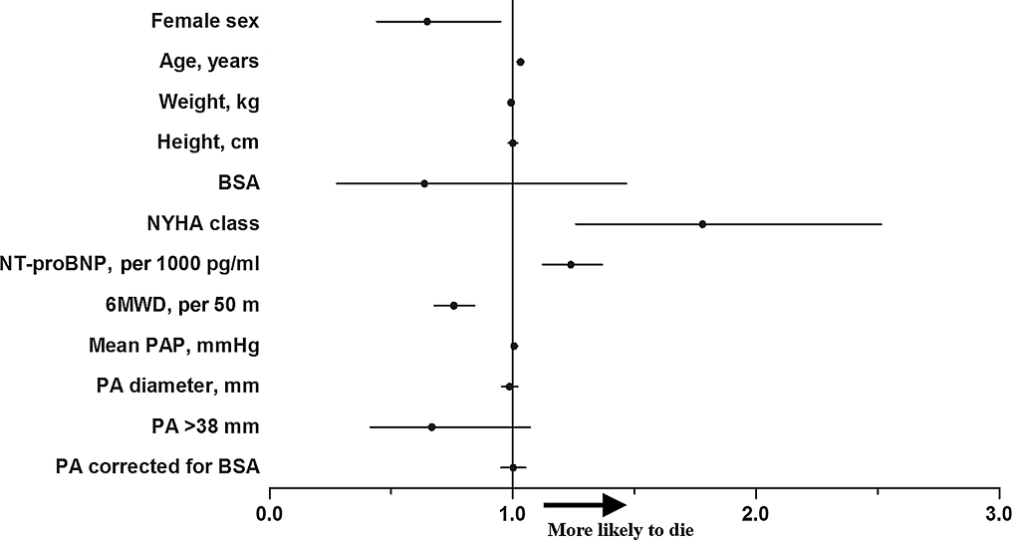
Forest plot showing the odds ratios for the association between the presence of a risk factor and the likelihood of dying. *BSA* body surface area, *NYHA FC* New York Heart Association functional class, *PA* pulmonary artery, *PAP* pulmonary artery pressure, *NT-proBNP* N-terminal pro-brain natriuretic peptide, *6MWD* 6-min walking distance

As illustrated in Fig. 4 (univariate analysis), in addition to the already known prognostic factors (6MWD, NT-proBNP, NYHA class), higher age and male sex were associated with worse outcome. PA diameter measured at diagnosis was not associated with mortality, even after correcting the diameter for BSA. A value for PA diameter above the third percentile (>38 mm) was also not associated with mortality. Multivariate analysis showed that female sex, lower age, longer 6MWD and lower NT-proBNP level were independent variables associated with better survival. In our cohort, the NYHA classification was not independently associated with mortality.

## Discussion

This study describes a large cohort with a long follow-up of patients with PAH and CTEPH. It was hypothesised that patients with a larger PA diameter at diagnosis would have a worse prognosis. This hypothesis could not be confirmed.

We found a poor cumulative survival of 87% at 1 year, 70% at 3 years and 58% at 5 years. Although new treatment options have become available in the Netherlands in recent years, we did not observe an increase in the survival rate of patients diagnosed in 2013–2018 compared to those diagnosed 5 years earlier.

As stated in the current European Society of Cardiology (ESC) PH guidelines, a PA diameter >25 mm is suggestive for the presence of PH [18]. However, some studies have shown that the upper limit of normal is 27 mm for women and 29 mm for men. In 95% of our study population, the PA diameter was above these predefined reference values. This supports the view that measuring the PA diameter can be useful for identifying patients with PH, as shown previously [7–9].

The hypothesis of this study was that the PA diameter was associated with the stage of the disease, and thus to prognosis, but this could not be confirmed. Most likely this is explained by the fact that in >95% of the patients the PA diameter was already dilated at presentation and there were simply not enough patients without PA dilatation. Whether or not the PA dilates further is not only pressure dependent. There could be several reasons why PA diameter was not associated with mortality. One explanation could be that after a certain moment in time PA dilatation decreases or stops. This is due to the fact that progression of the dilatation is different in every patient and is probably no longer only dependent on PAP. Lower PAP results in less wall tension. This is supported by the observation of a very weak correlation between PAP and PA diameter [13, 19].

The fact that there is a difference in PA diameter increase is potentially explained by the loss of distensibility, which among other reasons is probably age dependent, or due to a smaller surrounding space.

There is evidence from histological studies that dilatation is not only caused by changes in pressure, but by remodelling of the artery and alteration of the intrinsic vessel properties as well. These structural changes in collagen and elastin might by themselves cause dilatation, regardless of the height of the pressure or duration of PH. These processes differ between patients and may therefore be a factor leading to a higher or lower risk of a fatal outcome [20, 21].

Most patients in our study had advanced disease, illustrated by the high level of positive high-risk prognostic factors and the high risk score. As the disease progresses, the pulmonary vascular resistance increases and eventually leads to right ventricular failure. The right ventricle is not able to generate enough pressure, resulting in a decrease in pulmonary blood flow and pulmonary pressure. Since blood flow and pressure are important determinants of arterial dilatation and arterial remodelling, this might slow down the process of dilatation [19, 22].

PA diameter measurement was performed on different types of CT scans (with or without contrast, non-triggered, different slice thickness), which could have led to less comparability and larger measurement variation and could have led to the absence of an association between PA diameter and prognosis.

Several studies have tried to prove that PA diameter is related to mortality, but were not able to draw definitive conclusions because in these studies even already known risk factors did not have a significant correlation with mortality [12, 22]. In our study, we confirmed the associations of NT-proBNP and 6MWD as predictors of survival, but not for NYHA class. A possible explanation for this might be the low number of patients with a NYHA class ≤II, which makes it more difficult to use NYHA class as a predictor in this cohort.

One study found a relationship between unexpected death and severe PA dilatation [11], linking complications due to PA dilatation, including PA aneurysms, rupture and dissection or compression of the left main coronary artery leading to arrhythmias and unexpected death. As there was no association between PA dilatation and mortality in this study, we did not search for a relation between dilatation and specific causes of death. Badagliacca et al. [22] also found no association between PA size and mortality, confirming our results.

The risk score (the combination of NYHA functional classification, 6MWT and NT-proBNP level) was high at diagnosis, suggesting that patients were referred late in the course of the disease. The reasons for the late recognition of this rare disease are probably the lack of education for professionals, lack of awareness among patients and professionals and a lack of easily accessible and easy-to-use diagnostics.

This study found no significant difference in survival rates between patients diagnosed between 2013 and 2018 and those diagnosed in the period 2008–2013. However, it is not possible to draw decisive conclusions with our current data, since the patients diagnosed in the period after 2013 have not yet been followed up long enough, and the groups were not corrected for other factors, like use of PH-modifying medication. Considering that several new treatment options have become available with promising results, we expected to find an increase in survival [23]. Longer follow-up and further research are necessary to explore whether these new treatment options do increase survival in a real-life setting.

## Conclusion

Our findings suggest that PA diameter at diagnosis is not predictive for mortality in PAH and CTEPH patients. This study therefore suggests that PA diameter cannot be used for the prognosis of these patients. We observed a poor survival rate, especially in CTD-PH and IPAH patients. In the uncorrected survival comparison, no significantly increased survival rate was observed in the current era, which might be the result of the large proportion of CTD-PH patients with a known poor prognosis in this cohort. The very large proportion of patients with a high NYHA class at diagnosis raises the question of whether the disease could be identified earlier. Education of professionals and patients, together with improved diagnostics, might lead to an earlier referral and start of PH-modifying treatments, which could lead to improved survival.
